# *Plasmodium falciparum* malaria parasitaemia among indigenous Batwa and non-indigenous communities of Kanungu district, Uganda

**DOI:** 10.1186/s12936-016-1299-1

**Published:** 2016-05-04

**Authors:** Blánaid Donnelly, Lea Berrang-Ford, Jolène Labbé, Sabastian Twesigomwe, Shuaib Lwasa, Didacus B. Namanya, Sherilee L. Harper, Manisha Kulkarni, Nancy A. Ross, IHACC Research Team, Pascal Michel

**Affiliations:** Department of Geography, McGill University, Burnside Hall Building, 805 Sherbrooke St West, Montreal, QC H3A 0B9 Canada; Batwa Development Program, Buhoma, Kanungu District, Uganda; Department of Geography, Geoinformatics and Climatic Sciences, School of Forestry, Environmental and Geographical Sciences, CAES, Makerere University, P.O Box 7062, Kampala, Uganda; Ministry of Health, Plot 6 Lourdel Rd, P.O Box 7272, Kampala, Uganda; Department of Population Medicine, Ontario Veterinary College, University of Guelph, Guelph, ON N1G 2W1 Canada; School of Epidemiology, Public Health and Preventive Medicine, University of Ottawa, 600 Peter Morand Cres., 301E, Ottawa, ON K1H 8M5 Canada; Office of the Chief Science Officer, Public Health Agency of Canada, 130 Colonnade Road, Ottawa, ON K1A 0K9 Canada

**Keywords:** Indigenous health, Batwa, Malaria prevalence, Malaria risk factors, Livestock, Zooprophylaxis, Uganda

## Abstract

**Background:**

The indigenous Batwa of southwestern Uganda are among the most highly impoverished populations in Uganda, yet there is negligible research on the prevalence of malaria in this population. *Plasmodium falciparum* malaria parasitaemia prevalence was estimated in an indigenous Batwa and a non-indigenous neighbouring population, and an exploration of modifiable risk factors was carried out to identify potential entry points for intervention. Additionally, evidence of zooprophylaxis was assessed, hypothesizing that livestock ownership may play a role in malaria risk.

**Methods:**

Two cross-sectional surveys of Batwa and non-Batwa communities were carried out in Kanungu District, Uganda in July 2013 and April 2014 based on a census of adult Batwa and a two-stage systematic random sample of adult non-Batwa in ten Local Councils where Batwa settlements are located. A community-based questionnaire and antigen rapid diagnostic test for *P. falciparum* were carried out in the cross-sectional health surveys. A multivariable logistic regression model was built to identify risk factors associated with positive malaria diagnostic test. A subset analysis of livestock owners tested for zooprophylaxis.

**Results:**

Batwa experienced higher prevalence of malaria parasitaemia than non-Batwa (9.35 versus 4.45 %, respectively) with over twice the odds of infection (OR 2.21, 95 % CI 1.23–3.98). Extreme poverty (OR 1.96, 95 % CI 0.98–3.94) and having an iron sheet roof (OR 2.54, 95 % CI 0.96–6.72) increased the odds of infection in both Batwa and non-Batwa. Controlling for ethnicity, wealth, and bed net ownership, keeping animals inside the home at night decreased the odds of parasitaemia among livestock owners (OR 0.29, 95 % CI 0.09–0.94).

**Conclusion:**

A health disparity exists between indigenous Batwa and non-indigenous community members with Batwa having higher prevalence of malaria relative to non-Batwa. Poverty was associated with increased odds of malaria infection for both groups. Findings suggest that open eaves and gaps in housing materials associated with iron sheet roofing represent a modifiable risk factor for malaria, and may facilitate mosquito house entry; larger sample sizes will be required to confirm this finding. Evidence for possible zooprophylaxis was observed among livestock owners in this population for those who sheltered animals inside the home at night.

## Background

There are renewed calls for malaria eradication with a focus on Africa [1, 2]. Malaria mortality rates are decreasing in many populations, with global incidence having fallen by approximately 37 % since 2000 to 214 million new cases in 2015 [3, 4]. Despite these gains, malaria remains a major global disease burden, with approximately 438,000 deaths annually [3, 5].

The elimination of malaria from many western nations has been attributed primarily to social and economic development allowing for screening of windows and doors, destruction of vector breeding sites, and rapid diagnosis and treatment [6]. The feasibility of *Plasmodium falciparum* malaria elimination in most of sub-Saharan Africa is low, with Uganda being among the countries with lowest feasibility [7]. Sub-Saharan African countries are disproportionately affected by malaria [4] due to the presence of highly competent mosquito vectors, widespread poverty, limited infrastructure, and overburdened health systems [8, 9]. Those living in extreme poverty are most vulnerable to infectious diseases, yet within-country disparities are often ignored [10, 11]. African indigenous populations in particular have consistently poorer health outcomes than their non-indigenous counterparts [12]. Social determinants of indigenous health include, but are not limited to, poverty, discrimination, limited access to health care, and loss of traditional lands [13]. Indigenous and ethnic minority populations outside of Uganda experience higher rates of malaria, which have been attributed to relative impoverishment, marginalization, and geographic remoteness [14–18]. In some cases, genetic variations have been identified as drivers of ethnic differences in malaria parasitaemia and immunological response [19].

Risk factors for malaria can be conceptualized as non-modifiable and modifiable. Non-modifiable factors such as age and sex have been inconsistently associated with higher malaria risk. In endemic areas, children under 5 years of age have high risk of malaria due to their immunological naïveté [10, 20, 21]. Beyond this, the relationship between age and malaria in adults is less clear [22]. It is known that regular exposure to malaria results in a functional immunity to the disease which quickly wanes in the absence of exposure. Excluding immunologically-suppressed pregnant women, sex-related variations in malaria risk are generally linked to gender-role and occupational exposures [23, 24]. Women in roles as household water-collectors may spend more time near mosquito breeding sites. Men with forest-related jobs may spend their time in mosquito-dense areas [10, 23].

Modifiable risk factors include environmental conditions and human behaviour. Local vector ecology, including the locations of swamps, forests, rice paddies where vectors breed, and the proximity of these sites to human habitations may bring humans into frequent contact with mosquitoes [25–27]. Housing conditions may also affect transmission. Open eaves and windows, for example, may permit mosquito entry into sleeping quarters [25, 28, 29]. Education and wealth are known to be protective against malaria. Understanding malaria transmission and prevention may result in behaviours such as staying indoors during peak vector activity, and having access to preventive strategies that may reduce exposure of humans to vectors [27, 30]. Many of these risk factors are mediated by poverty whereby access to building materials and bed nets may be dependent upon income [31–33]. Poverty has been described as a key modifiable determinant of malaria burden [6].

Livestock are routinely included in analyses of risk factors for malaria infection, yet their role in malaria transmission is not well understood. Livestock are dead-end hosts for human-infectious *Plasmodium* parasites and may reduce human malaria risk by drawing vectors away from humans (zooprophylaxis) [34–36]. In some cases, however, livestock act as additional blood meal sources, and they may alter vector longevity and population density to increase human malaria risk (zoopotentiation) [37, 38]. Empirical research exploring the association between livestock and malaria risk has been complicated by the confounding role of wealth [39]. Livestock symbolize social standing, provide food and services, and act as an asset to be sold in times of financial need [40–44]. Both livestock and wealth are generally associated with lower malaria prevalence. Given efforts to include zooprophylaxis in integrated vector control programs, there have been calls for further research to understand the impact of livestock on malaria transmission [39].

The Ugandan Batwa are an indigenous population with life expectancy and child mortality rates significantly worse than national averages [45]. There remain considerable gaps regarding our understanding of Batwa health; to our knowledge only two peer-reviewed studies currently report the prevalence and risk factors of health outcomes for Batwa in Uganda [23, 46]. While literature on Batwa livelihoods is currently limited, it is thought that Batwa engage in livestock livelihoods at a much lower rate than non-Batwa due to financial restrictions, in addition to their historical hunter-gatherer culture. Further, Namanya identified consistent differences across risk factors for malaria, including housing and education between Batwa and non-Batwa [47].

The aims of this paper were (1) to estimate malaria prevalence in indigenous Batwa of Kanungu District and compare this with their non-indigenous neighbours, (2) explore modifiable risk factors for malaria parasitaemia in order to identify potential entry points for intervention to reduce malaria prevalence among Batwa and non-Batwa and, (3) to test the hypothesis of zooprophylaxis in a sub-set of livestock owners from both Batwa and non-Batwa populations.

## Methods

### Study population

Kanungu District is located in southwestern Uganda bordering the Democratic Republic of Congo to the west. It contains part of the Bwindi Impenetrable National Park (Bwindi) [48] and has a population of approximately 250,000, 80 % of whom live in rural settlements [49]. The majority of the population are of Bakiga ethnicity [45, 50]. The non-Batwa (non-indigenous populations, including Bakiga) rely mainly on subsistence farming consisting of cash- and food-cropping, and small-scale livestock holdings [48, 50], but tourism centred around gorilla trekking in Bwindi also provides local employment [45]. The indigenous Batwa population of Kanungu District is approximately 900 [51]. When Bwindi received its national park designation in 1991, the Batwa were no longer permitted to enter the park. Thus, they lost their ancestral lands and livelihood as traditional hunter-gatherers. Since then, the Batwa have been transitioning to settled agriculture [45]. Kanungu District is remote and has limited infrastructure and service delivery [50]. It receives rainfall throughout the year, with two dryer seasons from December to February and from June to July. Malaria in Kanungu is characterized by low transmission intensity and low endemicity [8, 23, 52, 53].

### Study design and sample

**Fig. 1:**
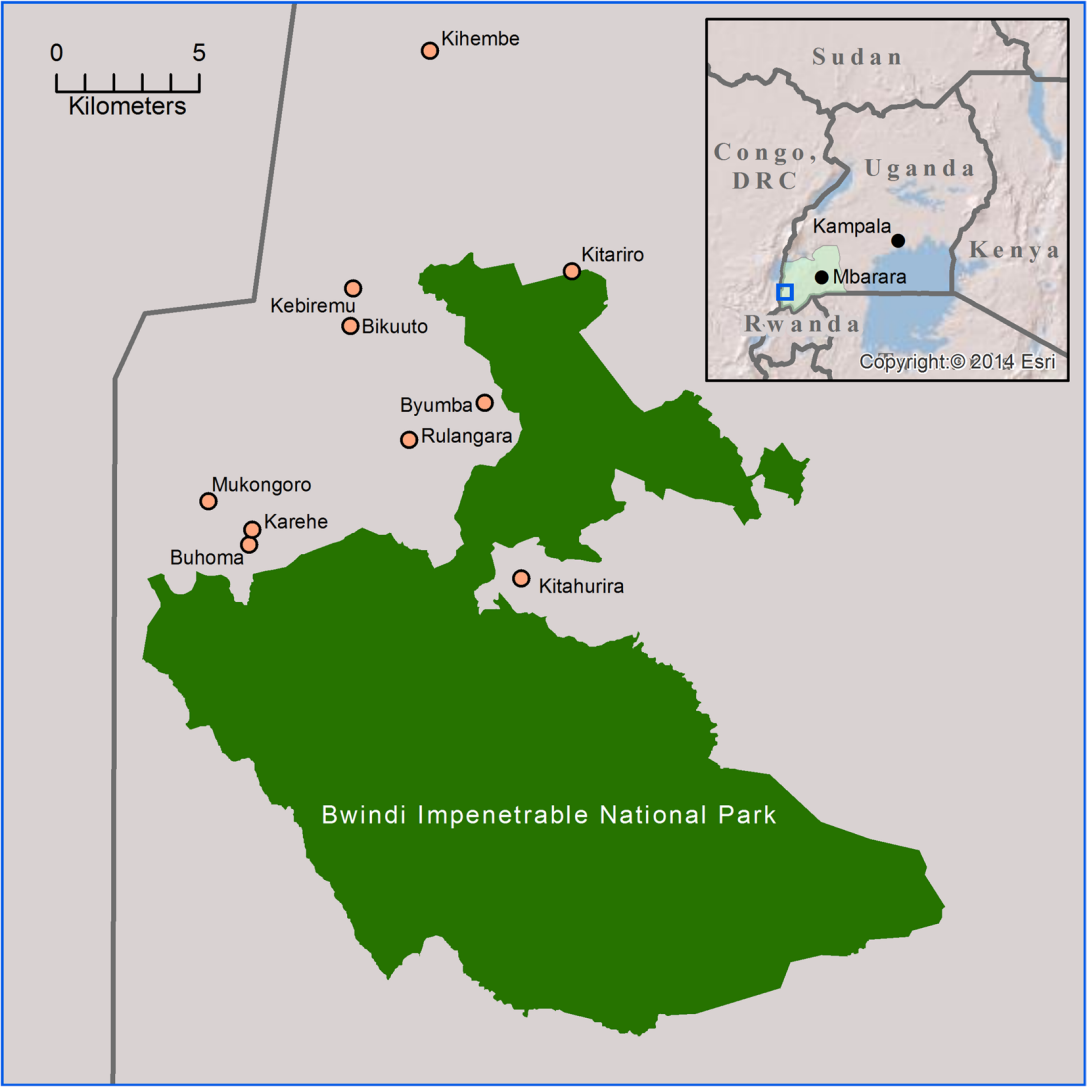
Kanungu District Batwa settlements surrounding Bwindi Impenetrable National Park, Uganda

Two cross-sectional, in-person surveys were administered in the 10 Local Councils (LCs, the smallest government administration unit in Uganda) containing the 10 Batwa settlements in July 2013 and April 2014 (Fig. 1). These 10 LCs are located within the subcounties of Kayantorogo, Kayonza, Kirima, Mpungu and Butogota Town Council. Considering the small total population size of the Kanungu Batwa population, a census of all adult Batwa present (>18 years) was attempted. A two-step proportional systematic random-sample of adult non-Batwa was conducted, representing approximately 40 % of non-Batwa households in each LC. Sampling frames (a list of households in the LC) were provided by Batwa settlement and non-Batwa Local Council 1 (LC1) chairpersons. The sampling strategy consisted of the following: a number between 1 and 10 was randomly selected as the first household to be selected from the sampling frame. The total number of households was multiplied by 0.4 to obtain the sampling interval required to obtain a 40 % sample of the LC. Every nth household was then selected from the sampling frame. For the second stage, the LC1 chairperson conducted a census of all adults living in the selected households from stage 1. The LC1 chairperson selected the adult to be sampled out of a hat based on the census of adults in those households. The same individuals were interviewed in July and April.

### Data collection

*Plasmodium falciparum* is the primary cause of malaria in this region [8]. Parasitaemia (outcome variable) was classified as a positive result on *CareStart*™ malaria rapid diagnostic test (RDT) detecting histidine-rich protein 2 (HRP2) of *P. falciparum* (Access Bio, Inc. NJ, USA) at either the July or April data collections [54]. The RDT has a sensitivity of 98 % and specificity of 97.5 % [55]. Having a positive test result at either collection period was defined as “RDT ever” while a negative outcome at both collection periods was defined as “RDT never”.

At each survey (July and April), participants were interviewed in Rukiga (the local language for Batwa and non-Batwa) by trained local survey administrators. The survey consisted of three parts: (1) an individual-level health questionnaire, (2) a household questionnaire and (3) a malaria RDT administered by a community health nurse. Part one (individual questionnaire) collected information pertaining to demographics and personal information, health and risk behaviour, and livelihoods. Part two (household questionnaire) collected information on household structure, housing materials, assets, water, and sanitation. For Batwa, each individual completed parts one and three, with the household head completing part two. The individual questionnaires were later matched to the household questionnaires. For non-Batwa, each individual completed all components. Data were collected in hard copy and entered into Microsoft Access (Microsoft Corp, USA).

### Ethics

This study was designed using a community-based participatory research approach and was approved by the Research Ethics Boards at McGill University and the University of Guelph. The study design is consistent with the Canadian Tri-Council’s Policies and requirements of the Ethical Conduct of Research Involving Human Subjects. Informed consent was obtained verbally from all participants. This study is a part of the Indigenous Health Adaptation to Climate Change (IHACC) project, an international initiative with parallel field sites in the Canadian Arctic and Peruvian Amazon [56].

### Statistical analysis

#### Malaria parasitaemia prevalence and risk factors

Among Batwa and non-Batwa a total of 649 questionnaires were carried out in July 2013 and 546 surveys were completed in April 2014. All questionnaires from July and April were pooled to create a single study population. This was done to: (1) increase the sample size, (2) achieve a prevalence reflective across seasons, and (3) account for the potential sporadic nature of infection and risk factors using only one point estimate in time. For individuals who were present for both the July and April surveys, and who were RDT negative at both times (n = 709), the risk factor data from the July surveys were used as baseline. If an individual was present in July and April and was RDT positive at one time (n = 49), data from the time at which they tested positive were used. If they were present at both July and April and positive at both times (n = 1), the July data were used. The period prevalence was calculated by dividing the total number of positive cases by the total number of participants tested.

To identify risk factors associated with parasitaemia, a series of univariable logistic regression models were constructed. Risk factors of interest were selected based on theorized relevance in the literature and data availability. Covariates included: age, sex, knowledge of malaria transmission (as reflected by knowing that one should avoid mosquito bites to prevent malaria), roofing material, bed net ownership, and wealth (Table 1). To measure wealth, a household-level asset-based wealth index was created using principal component analysis [46, 57, 58]. The variables included in the index were cell phone ownership, radio ownership, bicycle ownership, having a source of electricity, land ownership, receiving remittances, toilet type, and having access to hand washing facilities. The resultant component was categorized as a dichotomous variable representing the 50 % least poor relative to the 50 % poorest individuals. Livestock were not included in the wealth index to allow the evaluation of their specific role in malaria transmission. Collinearity between risk factor variables was assessed using a Spearman’s rank correlation coefficient with a cut-off of 60 %. Where collinearity was identified, the variable with the strongest association with the outcome variable was retained. A multivariable logistic regression model was built, and a final, reduced multivariable model based on variables significant in either the univariable or multivariable model. A mixed-effects logistic regression model with random intercept was tested, but there was no evidence of clustering at the LC-level. Data were analysed using Stata v.11 (Stata Corp., USA).

**Table 1:** Variables relating to malaria risk factors for Batwa and non-Batwa communities in Kanungu District, Uganda

Construct	Description	Variable	Justification
Dependent variable			
Malaria parasitaemia *(P. falciparum)*	Positive RDT July 2013 Positive RDT April 2014	Dichotomous (ever positive/never positive)	RDTs are a widely accepted method of malaria detection and may outperform microscopy in some settings [4, 80–82]
Independent variables			
Ethnicity	Survey date	Dichotomous dummy variable (Batwa/non-Batwa)	Indigenous populations and ethnic minorities have been identified in the literature as being more vulnerable to malaria [14–18]
Livestock ownership	Household ownership of animals (any)	Dichotomous (yes/no)	Livestock have been associated with increases in malaria prevalence through zoopotentiation [25, 38], and decreases in prevalence through zooprophylaxis [76, 77, 83] and wealth [26, 33]
Control variables			
Individual-level			
Sex	Observed sex of participant	Dichotomous (male/female)	Sex is associated with increased risk due to variations in exposure through livelihood or household activities [23, 24]
Age	Self-reported age of participant	Continuous (years)	Children, in areas of high transmission, are more vulnerable to infection than adults due to a lack of acquired immunity [10, 20, 21]
Malaria-related knowledge	Question: What is the best way to prevent malaria?	Dichotomous dummy variable based on correct identification of avoiding mosquito bites.	Understanding malaria transmission is associated with preventive behaviours [10, 84, 85]
Bed net use	Question: Does your household own a mosquito net?	Dichotomous (yes/no)	Insecticide treated and untreated bed nets are associated with significant reductions in malaria prevalence [21, 84, 85]
Household-level			
Wealth	Question: Does your household have any of the following items?	Dichotomous dummy variable based on categorization of PCA of variables: cell phone, radio, bicycle, electricity, private latrine, hand washing facilities, remittance, land (lowest 50 % of scores/highest 50 % of scores)	Wealth is associated with access to malaria prevention and health care [10, 31, 84, 85]
House construction	Question: What is your roof made of?	Dichotomous (iron sheets/wood or thatch or banana fibre)	Wall materials, open eaves, and window coverings can facilitate vector entry into the home [24, 28, 29]
Animals kept inside at night	Question: If you own animals, do your animals come into the house during the night?	Dichotomous dummy variable (yes/no)	Zooprophylaxis/zoopotentiation may be determined by relative proximity of animals to human sleeping quarters [29, 86–88]

### Zooprophylaxis

A subset analysis of livestock owners assessed the impact of relative location of livestock to humans. The subset was achieved by selecting from the survey dataset all Batwa and non-Batwa individuals who had a positive outcome on the “any livestock” variable. Among those who owned any livestock, the risk factor of interest was whether livestock were housed inside or outside of the home at night. This analysis controlled for bed net use and non-livestock wealth, which have been proposed as potential confounding and effect modifying variables, respectively [39]. Sensitivity analyses were used to assess the effect of housing specific livestock species (goats and chickens) inside the house on parasitaemia. Although age and sex were not significant in the reduced multivariable model, sensitivity analysis also tested their potential effect on the final model.

## Results

### Batwa and non-Batwa demographics

A total of 759 individual questionnaires were completed in July 2013 and April 2014, of which 59.10 % (449) of participants were non-Batwa and 40.90 % (310) Batwa (Table 2). Females were overrepresented in the study population compared to males, slightly more so among non-Batwa than among Batwa. Men were more likely than women to be employed or away finding work and therefore unavailable to participate. The population had an average age of 36.6 years with the Batwa being slightly younger on average than non-Batwa (34.9 and 37.6 years, respectively, z 2.88, p < 0.01). Educational attainment differed significantly between indigenous and non-indigenous with 41.78 % of Batwa having any education compared to 73.21 % of non-Batwa (χ^2^ 77.23, p < 0.01). About 40 % of adults were employed with no significant difference between groups (χ^2^ 0.39, p 0.53). 70.55 % of Batwa were in the low wealth category compared to 38.07 % of non-Batwa (χ^2^ 73.81, p < 0.01). While there was no difference in the average number of household members between the two ethnic groups (Batwa 5.16 people/household, non-Batwa 5.0 people/household, p 0.73), Batwa experienced a greater level of crowding than non-Batwa (2.81 individuals/sleeping room and 2.54 individuals/sleeping room, respectively, z −2.75, p < 0.01). Bed net ownership (50.49 % Batwa versus 60.09 % non-Batwa, χ^2^ 6.84 p < 0.01) and use were lower among Batwa than non-Batwa (38.61 and 58.07 % slept under a bed net the previous night respectively, χ^2^ 27.32, p < 0.01). Livestock ownership was consistently lower among Batwa than non-Batwa with 32.04 % of Batwa owning any livestock compared to over half of non-Batwa (χ^2^ 35.68, p < 0.01). Goats were the most frequently owned livestock among non-Batwa (16.13 % of Batwa and 41.22 % of non-Batwa owned goats, χ^2^ 53.81, p < 0.01) while Batwa most frequently owned chickens compared to other animals but still, at a lower frequency than non-Batwa (18.71 % Batwa owned chickens compared to 31.53 % non-Batwa, χ^2^ 15.49, p < 0.01). Both Batwa and non-Batwa brought animals indoors at night, to protect against predation and theft (21.50 % Batwa, 22.90 % non-Batwa, χ^2^ 0.19, p 0.66).

**Table 2:** Demographics and parasitaemia status of Batwa and non-Batwa of Kanungu District, Uganda, July 2013 and April 2014

Characteristic	Total surveyed participants	RDT never	RDT ever
	n (%)	n (%)	n (%)
Individual characteristics			
Sex	n = 749	n = 701	n = 48
Female	452 (60.35)	423 (60.34)	29 (60.42)
Male	297 (39.65)	278 (39.66)	19 (39.58)
Age	n = 688	n = 654	n = 43
Mean	36.60 [95 % CI 35.44–37.8]	36.40 [95 % CI 35.21–37.50]	39.90 [95 % CI 33.50–46.20]
Ethnic group	n = 758	n = 709	n = 49
Non-Batwa	448 (59.10)	428 (60.37)	20 (40.82)
Batwa	310 (40.90)	281 (39.63)	29 (59.18)
Malaria prevention knowledge (avoid bites)	n = 755	n = 706	n = 49
No	588 (77.88)	545 (77.20)	43 (87.76)
Yes	167 (22.12)	161 (22.80)	6 (12.24)
Household characteristics			
Roof type	n = 752	n = 703	n = 49
Metal/iron sheets	621 (82.58)	577 (82.08)	44 (89.80)
Grass/thatch/wood	131 (17.42)	126 (17.92)	5 (10.20)
Own mosquito net	n = 755	n = 706	n = 49
No	331 (43.84)	306 (43.34)	25 (51.02)
Yes	424 (56.16)	400 (56.66)	24 (48.98)
Relative wealth	n = 728	n = 680	n = 49
Poorer 50 %	372 (51.10)	338 (49.71)	34 (70.83)
Wealthier 50 %	356 (48.90)	342 (50.29)	14 (29.17)
Livestock ownership			
Any livestock	n = 753	n = 704	n = 49
No	414 (54.26)	382 (54.26)	32 (65.31)
Yes	339 (45.02)	322 (45.74)	17 (34.69)
Chickens	n = 754	n = 705	n = 49
No	556 (73.74)	515 (73.05)	41 (83.67)
Yes	198 (26.26)	190 (26.95)	8 (16.33)
Goats	n = 754	n = 705	n = 49
No	521 (69.10)	479 (67.94)	42 (85.71)
Yes	233 (30.90)	226 (32.06)	7 (14.29)
Animals inside at night	n = 686	n = 637	n = 49
Never/no animals	533 (77.70)	487 (76.45)	46 (93.88)
Sometimes/often	153 (22.30)	150 (23.55)	3 (6.12)

### Malaria parasitaemia prevalence and risk factors

Of the 759 questionnaires completed in July 2013 and April 2014, 50 participants tested positive for *P. falciparum* antigen on RDT. One participant tested positive at both data collection events. Only the July data were considered for this participant, resulting in a total of 49 positive cases. Of the positive cases, 29 (59.18 %) were Batwa and 20 (40.82 %) were non-Batwa. The parasite period prevalence was 9.35 % and 4.45 % for Batwa and non-Batwa, respectively, thus the odds of a positive RDT result were 2.21 times higher for Batwa than for non-Batwa (95 % CI 1.23–3.98).

### Univariable analyses

Among the non-modifiable risk factors evaluated for Batwa and non-Batwa participants, individuals over 50 years of age appeared to be at increased odds (OR 1.49, 95 % CI 0.73–3.04) for malaria parasitaemia (relative to younger individuals), although this result was not precise, with wide confidence intervals (Table 3). Among modifiable risk factors for malaria, relative wealth had the strongest association with parasitaemia. Relative poverty was associated with higher odds of parasitaemia, having 2.48 times the odds compared to the wealthier individuals (95 % CI 1.30–4.66). Not owning a bed net, and iron sheet roofing compared to thatched roofs increased the odds of parasitaemia, although these estimates were imprecise. Not owning livestock (OR 1.59, 95 % CI 0.87–2.91), and not understanding the importance of avoiding mosquito bites (OR 2.11, 95 % CI 0.89–5.06) increased odds of positivity for malaria parasitaemia; again, however, estimates were imprecise with wide confidence intervals.

**Table 3:** Univariable and multivariable explanatory logistic regression analyses of malaria parasitaemia for Batwa and non-Batwa of Kanungu District, Uganda, July 2013 and April 2014

Risk factor	Univariable models Unadjusted odds (OR [CI])^a^	Reduced multivariable model Adjusted odds (OR [CI])^a^
		N = 717
Non-modifiable		
Age	N = 688	
<50 years	Ref	
≥50 years	1.49 [0.73–3.04]	
Sex	N = 749	
Female	Ref	
Male	0.99 [0.55–1.81]	
Ethnicity	N = 758	
Non-Batwa	Ref	Ref
Batwa	2.21 [1.23–3.98]	1.88 [1.00–3.58]
Modifiable		
Relative wealth	N = 728	
Wealthier 50 %	Ref	Ref
Poorer 50 %	2.48 [1.30–4.66]	1.96 [0.98–3.94]
Bed net ownership	N = 755	
Yes	Ref	
No	1.36 [0.76–2.43]	
Livestock ownership	N = 753	
Yes	Ref	Ref
No	1.59 [0.87–2.91]	1.44 [0.75–2.79]
House construction (roof)	N = 752	
Thatch	Ref	Ref
Iron sheets	1.92 [0.75–4.94]	2.54 [0.96–6.72]
Malaria knowledge (avoid mosquito bites)	N = 755	
Yes	Ref	Ref
No	2.11 [0.89–5.06]	1.92 [0.79–4.63]

^a^Unadjusted odds represents the logistic regression model for each variable alone and the outcome of interest (RDT positivity). Adjusted odds is the odds ratio when all listed variables are included in the logistic regression model. These results are consistent on sensitivity analysis for survey date

### Reduced multivariable model

In reduced multivariable analyses Batwa ethnicity (OR 1.88, 95 % CI 1.00–3.58), poverty (OR 1.96, 95 % CI 0.98–3.94), and iron sheet roofing (OR 2.54, 95 % CI 0.96–6.72) had increased odds of positive RDT. There was no association between LC and parasitaemia, nor was there a significant difference between models that controlled for clustering at the LC-level and those that did not. There was minimal difference in the fit of the model with and without random intercepts. The model results were not sensitive to survey date, and showed consistent results on the removal of ethnicity and/or wealth.

### Zooprophylaxis

Amongst the 339 Batwa and non-Batwa livestock owners within the total survey population of 759 respondents, 17 individuals had a positive RDT outcome. Of the 339 livestock owners, 99 (29.2 %) were Batwa and 240 (70.8 %) were non-Batwa. Keeping animals inside the house at night was significantly protective against malaria (OR 0.29, CI 0.09–0.94) in adjusted analyses (Table 4). The sample size was insufficient to disaggregate by livestock species (chickens and goats). Sensitivity analyses indicated consistency of results across species, as well as no effect on the results when adding age and/or sex in the model.

**Table 4:** Multivariable logistic regression analysis of malaria risk for Batwa and non-Batwa livestock owners (zooprophylaxis) in Kanungu District Uganda, July 2013 and April 2014

Variables	Adjusted odds (OR [CI])
	n = 325
Variable of interest	
Keep animals inside at night	
No	Ref
Yes	0.29 [0.09–0.94]
Control variables	
Ethnicity	
Non-Batwa	Ref
Batwa	1.79 [0.59–5.38]
Relative wealth	
Wealthier 50 %	Ref
Poorer 50 %	3.39 [1.06–10.85]
Bed net ownership	
Yes	Ref
No	0.88 [0.29–2.74]

Adjusted odds is the odds ratio when all listed variables are included in the logistic regression model. These results are not sensitive to age and sex on sensitivity analysis

## Discussion

The period prevalence of *P. falciparum* parasitaemia for July 2013 and April 2014 for adult Batwa was 9.35 %, which is higher than the 4.1 % rate previously reported for Batwa over the age of 5 years [23]. A bed net distribution was carried out in November 2012, which could explain the low prevalence rate found previously in this population in January 2013 [59]. These findings are consistent with parasite prevalence estimates for Kanungu District as being relatively low (10–40 % prevalence when including children) compared to other parts of Uganda [8, 60].

Ethnicity was associated with malaria parasitaemia, with Batwa at higher risk. This is an important result given that wealth and other theorized risk factors for parasitaemia were controlled for; there remains a residual effect of ethnicity beyond these factors. There are marked differences in livelihoods between the two populations that may be driving these results. Batwa are currently undergoing a transition as they adapt to life outside of the forest. This transition is reflected in the low levels of education and livestock ownership relative to non-Batwa. There may also be unmeasured genetic differences between these ethnic groups that influence malaria prevalence [61–64]. For instance, ethnic differences in immune responses [19, 65] and lower susceptibility to malaria infection among ethnic minority Fulani tribes relative to sympatric ethnic groups in West Africa have been attributed to genetic differences at various loci [66, 67]. It might be expected, given their higher prevalence of infection, that Batwa mount a weaker immune response relative to non-Batwa in the face of exposure. Immunological studies would be required to test this hypothesis, but it is unclear how the results of such studies would lead to intervention strategies [6].

These results provide tentative indication that there may be important modifiable risks for malaria infection among Batwa and non-Batwa. Iron sheet roofing may play a role in malaria risk. House construction is an important risk factor for malaria infection [68–70] and thatched houses have previously been related to higher malaria prevalence of inhabitants in other parts of sub-Saharan Africa [28] due to the propensity of mosquitoes to rest indoors [25]. However, a systematic review and meta-analysis on the effect of house construction on malaria found that modern roof materials (iron sheets, tiles) were not consistently associated with decreased odds of infection [71]. Thatched or wooden roofs may confer some protection to individuals relative to corrugated iron sheets. It is possible that the open eaves of iron sheet roofs facilitate mosquito house entry and thereby increased malaria risk. Open eaves and gaps in housing materials are frequently associated with higher rates of parasitaemia [24, 29, 71]. However, this result may be confounded by wealth. Among non-Batwa, iron sheet roofs are a reflection of wealth since they must be purchased at a high cost compared to thatch or banana fibre, which may be obtained at no cost from the surrounding environment. In contrast, iron sheet roofing is purchased for Batwa by a local NGO with priority given to the most impoverished families as identified by the community. A Ugandan study carried out in part in Kanungu District found that closed eaves and modern house construction were associated with significantly decreased human biting rates and incidence rates of malaria in children when controlling for age, sex, and household wealth [72]. Further evaluation of the impact of house construction on malaria risk among Batwa may help to inform and improve these community development strategies.

The analysis of livestock owners suggested that when controlling for wealth and bed net use, keeping animals inside at night reduced the odds of malaria infection. Previous studies suggest that keeping animals indoors at night increases malaria risk where mosquitoes are zoophilic [39]. Entomological studies in the region suggest that *Anopheles funestus* and *Anopheles gambiae* sensu lato are the predominant vector species [60]. *Anopheles funestus* tends towards anthropophily; however, *An. gambiae* s.l. consists of seven morphologically indistinguishable species [73] of which the most important vectors may be more zoophilic, as with *Anopheles arabiensis*, or anthropophilic, as with *An. gambiae* sensu stricto. This finding might suggest that the predominant local species are anthropophilic and, in turn, that keeping animals indoors at night results in reduced malaria risk among livestock owners, however further entomological evaluation of local vector populations is required. The mechanism through which livestock infer protection against malaria remains poorly understood and their role in malaria transmission has been much contested [32, 35, 37, 38, 74, 75]. Livestock may draw mosquitoes away from humans, reducing their exposure to malaria [76, 77], or may provide an abundance of blood-meals, increasing vector density and longevity [32, 38, 74]. The zooprophylatic effect is, however, complicated by the relationship between livestock and wealth [32, 39]. It is well recognized that livestock contribute significantly to household economies for the rural poor, including the most marginalized [40, 41, 43]. Wealthy community members in Kanungu are those with the greatest livestock and land holdings. The findings of this study are consistent with others showing that poverty is positively associated with malaria prevalence [31, 78, 79].

The cross-sectional nature of this study limits the ability to infer causal relationships between the risk factors and malaria infection. Unemployed people who were available to be surveyed during data collection events were overrepresented in the sample. This may have reduced variation within the population and perhaps led to an underestimation of the importance of employment for malaria infection. Similarly, men were more likely to be working away, leading to an overrepresentation of women. Given that the role of sex in malaria risk remains unclear, it is difficult to predict the direction of this effect. The small sample size and number of cases limited statistical power to detect effect sizes. Sample size also prevented the stratification of uni- and multi-variable analyses of parasitemia based on ethnicity, although sensitivity analysis suggested results were consistent between ethnic groups. Similarly, sample size prevented ethnicity-based stratification for the zooprophylaxis analysis. As a result, the identification of risk factors, and recommendations for malaria control, apply to the survey population as a whole. In highly vulnerable remote indigenous populations, sample sizes are typically small and demographics frequently differ from non-indigenous populations; lack of statistical power must be balanced with the importance of prioritizing research within vulnerable and at-risk sub-populations.

## Conclusions

Indigenous Batwa in Kanungu District, Uganda experience a two-fold increase in malaria risk compared to their non-indigenous neighbours. This inequitable burden mirrors health disparities experienced by indigenous peoples worldwide. Tentative support for the role of housing construction and wealth in accounting for some of the risk differential was found. Investigation of roof construction and related vector entry may be a prudent follow-up measure given these results. High baseline poverty among both populations, but particularly among Batwa, will remain a major determinant of health inequity and transmission risk. The influence of livestock may be related to asset-based wealth and suggests that keeping livestock indoors at night may play a role in reducing human exposure to malaria in this setting. This evidence may support livestock-based interventions here as both a poverty reduction strategy as well as a component of malaria vector control.

## Abbreviations

RDT: rapid diagnostic test
LC: Local Council
